# The Association Between Metformin Treatment and Outcomes in Type 2 Diabetes Mellitus Patients With Heart Failure With Preserved Ejection Fraction: A Retrospective Study

**DOI:** 10.3389/fcvm.2021.648212

**Published:** 2021-03-12

**Authors:** Jianfang Wang, Yi Lu, Xinjia Min, Tan Yuan, Jia Wei, Zhejun Cai

**Affiliations:** ^1^Department of Cardiology, The Second Affiliated Hospital, Zhejiang University School of Medicine, Hangzhou, China; ^2^Ningbo Medical Center Lihuili Hospital, Ningbo, China; ^3^Department of Urology, Children's Hospital, Zhejiang University School of Medicine, Hangzhou, China; ^4^Jiaxing Key Laboratory of Cardiac Rehabilitation, Jiaxing, China

**Keywords:** metformin, heart failure with preserved ejection fraction, type 2 diabetes mellitus—exenatide, survival analysis (source: MeSH NLM), mortality

## Abstract

**Background:** Metformin is the first-line antidiabetic medication for type 2 diabetes mellitus (T2DM). However, the association between metformin and outcomes in T2DM patients with heart failure with preserved ejection fraction (HFpEF) is still unknown. We aimed to explore the association between metformin and adverse outcome in T2DM patients with HFpEF.

**Methods:** A total of 372 T2DM patients with HFpEF hospitalized from January 1, 2013, to December 31, 2017, were included in this retrospective cohort study. There were 113 and 259 subjects in metformin and non-metformin group, respectively. Subjects were followed up for all-cause mortality, cardiovascular death, all-cause hospitalization, and heart failure hospitalization.

**Results:** The median follow-up period was 47 months. Eleven patients (2.49% per patient-year) in the metformin group and 56 patients (5.52% per patient-year) in the non-metformin group deceased during follow-up (*P* = 0.031). However, a multivariable Cox regression failed to show that metformin was an independent factor of all-cause mortality [HR (95% CI) = 0.682 (0.346–1.345); *P* = 0.269]. A subgroup analysis revealed a significant association between metformin and all-cause mortality in patients with a higher hemoglobin A1c (HbA1c) level (HbA1c ≥7%) [HR (95% CI) = 0.339 (0.117–0.997); *P* = 0.045]. The 4-year estimated number needed to treat (NNT) with metformin compared with non-metformin for all-cause mortality was 12 in all populations and 8 in the HbA1c ≥7% subgroup.

**Conclusions:** Metformin was not independently associated with clinical outcomes in patients with T2DM and HFpEF, but was associated with lower all-cause mortality in the subgroup of patients with poor glycemic control. Prospective, randomized controlled trials are needed to further verify these findings.

## Introduction

Heart failure with preserved ejection fraction (HFpEF) might be a heterogeneous syndrome of multiple discrete phenotypes and is prone to have multiple comorbidities, such as diabetes, hypertension, pulmonary disease, chronic kidney disease, and obesity (1), resulting in systemic and cardiac microvascular dysfunction (2, 3). Conventional therapies including angiotensin-converting enzyme inhibitors (ACEIs), angiotensin receptor blockers (ARB), beta-blockers, mineralocorticoid receptor antagonists (MRA) can improve the long-term outcomes of heart failure with reduced ejection fraction (HFrEF) (4). However, these conventional medical therapies failed to reduce the risk of all-cause and cardiovascular death in HFpEF patients (5).

Type 2 diabetes mellitus (T2DM) is a common comorbidity in HFpEF and has a conspicuous negative impact on prognosis (6). As first-line antidiabetic therapy, metformin has cardiovascular protective effect through multiple mechanisms, including decreasing glucose, lowering weight, anti-inflammatory properties, and improving insulin resistance and endothelial function (7). Several observational studies indicated that metformin was associated with reduced mortality risk compared with other traditional antidiabetic drugs in T2DM patients with HF (patients with preserved or reduced left ventricular ejection fraction were included) (8). However, the impact of metformin on the outcome of HFpEF in T2DM patients has not been elucidated. Therefore, we performed a retrospective cohort study to investigate the association between metformin and this specific group of patients suffering from HFpEF with T2DM.

## Materials and Methods

### Study Population

This is a retrospective cohort study conducted among in-hospital HFpEF (4) with T2DM patients admitted in the Department of Cardiology, the Second Affiliated Hospital, Zhejiang University School of Medicine from January 1, 2013, to December 31, 2017. The main inclusion criteria were (1) ≥40years of age; (2) had a left ventricular ejection fraction (LVEF) ≥50% and New York Heart Association (NYHA) class II to IV symptoms; (3) elevated B-type natriuretic peptide (BNP) ≥35 pg/mL or N-terminal pro-B-type natriuretic peptide (NT-proBNP) ≥125 pg/mL; (4) echocardiographic evidence of relevant structural heart disease (left atrial enlargement or left ventricular hypertrophy) or diastolic dysfunction (meet at least three of the following criteria simultaneously: left atrial enlargement, tricuspid regurgitation peak velocity >2.8 m/s, septal e' <7 cm/s or lateral e' <10 cm/s, E/e' >14), or imaging findings of pulmonary congestion; (5) diagnosed as T2DM in medical records and remained antidiabetic drugs therapy regularly for at least 3 months.

Patients were excluded if they: (1) had cardiovascular disorders that may change their clinical course independently of heart failure (such as myocardial infarction, coronary artery bypass graft surgery, or other major cardiovascular surgery, stroke or transient ischemic attack in the past 90 days); (2) currently implanted left ventricular assist device, or cardiac resynchronization therapy; (3) had a history of acute decompensated heart failure within 1 week of screening; (4) had a specific heart failure etiologies including hypertrophic obstructive cardiomyopathy, amyloidosis, acute myocarditis, pericardial disease, primary valvular heart disease requiring surgery or intervention, or severe conduction disorders requiring pacemaker implantation; (5) were previously diagnosed of reduced left ventricular EF <40%; (6) significant impaired renal function (estimated glomerular filtration rate of <45 mL/min/1.73 m^2^ measured by the CKD-EPI equation or requiring dialysis at the time of screening) or hepatic function; (7) short life expectation; (8) loss of follow-up; (9) metformin users who failed to taking metformin continuously.

### Data Collection

The demographic characteristics, underlying diseases, laboratory reports, antidiabetic therapy, and nursing records of study patients were collected. Demographic characteristics included age, gender, body mass index (BMI), cigarette smoking, blood pressure, heart function classification, left ventricular ejection fraction. Main underlying diseases included hypertension (HTN), atrial fibrillation (AF), coronary heart disease (CHD), or cerebral infarction (CI). Main laboratory reports included BNP, NT-proBNP, hemoglobin A1c (HbA1c), hemoglobin, serum creatinine, low-density lipoprotein cholesterol (LDL-C), high-density lipoprotein cholesterol (HDL-C), and triglyceride (TG). The CKD-EPI equation was adopted for estimating the glomerular filtration rate (eGFR) (9).

### Outcomes

Clinical endpoints were obtained through a median follow-up of 47 months *via* the EMR database and telephone connection. The primary endpoint was all-cause mortality. Secondary endpoints were cardiovascular death, all-cause hospitalization, and hospitalization for heart failure.

### Statistical Analysis

Baseline data were expressed as means ± standard deviation or median (25th and 75th percentiles). Student's *t*-test or Mann–Whitney-test was used to compare continuous variables between the two groups. Data were presented as the number (percentage), and the χ^2^-test was used to compare qualitative variables. The association between metformin and clinical outcomes was analyzed by Kaplan-Meier analysis. A multivariable Cox regression was performed using the stepwise regression, with a threshold of 0.1, to assess the independence of this association. Adjusted confounders included age, gender, body mass index, cigarette smoking, systemic blood pressure, diastolic blood pressure, left atrial diameter, left ventricular ejection fraction, NYHA class, duration of diabetes and heart failure, comorbidities including hypertension, atrial fibrillation, coronary heart disease, and cerebral infarction, laboratory findings including glycated hemoglobin, estimated glomerular filtration rate, hemoglobin, low-density lipoprotein cholesterol, high-density lipoprotein cholesterol and triglyceride, and usage of sulfonylureas, glinides, glucosidase inhibitors, and insulin.

Further, we explored the association between metformin and the primary outcome in selected subgroups. Multivariable Cox regressions with the same way and adjustments as the above were used in the subgroup analysis. The best glycaemic targets are still unclear, but most agree on HbA1c thresholds <7.0% for the majority of adults with DM. We categorized the subjects as HbA1c <7% and HbA1c ≥7% and did a subgroup analysis among patients with higher HbA1c levels. Major imbalances were found in age, sex, eGFR, and GI treatment between metformin and non-metformin users in the HbA1c ≥7% subgroup. We performed propensity score matching (PSM) of age and sex for patients in two groups. Matching was performed using the nearest neighbor matching, with a default caliper of 0.1.

The number needed to treat (NNT) values for metformin therapy incremental to non-metformin treatment were estimated for years 1 to 4 for the primary endpoint. NNT values were estimated as the inverse of the difference in estimated absolute risk between the metformin and non-metformin groups at each time point. The absolute risk for the non-metformin group was calculated directly from Kaplan-Meier estimates.

A two-sided *P* < 0.05 was considered statistically significant. The statistical analysis was performed using IBM SPSS Statistics for Windows, version 22.0 (IBM, Armonk, New York).

## Results

### Baseline Characteristics

**Figure 1:**
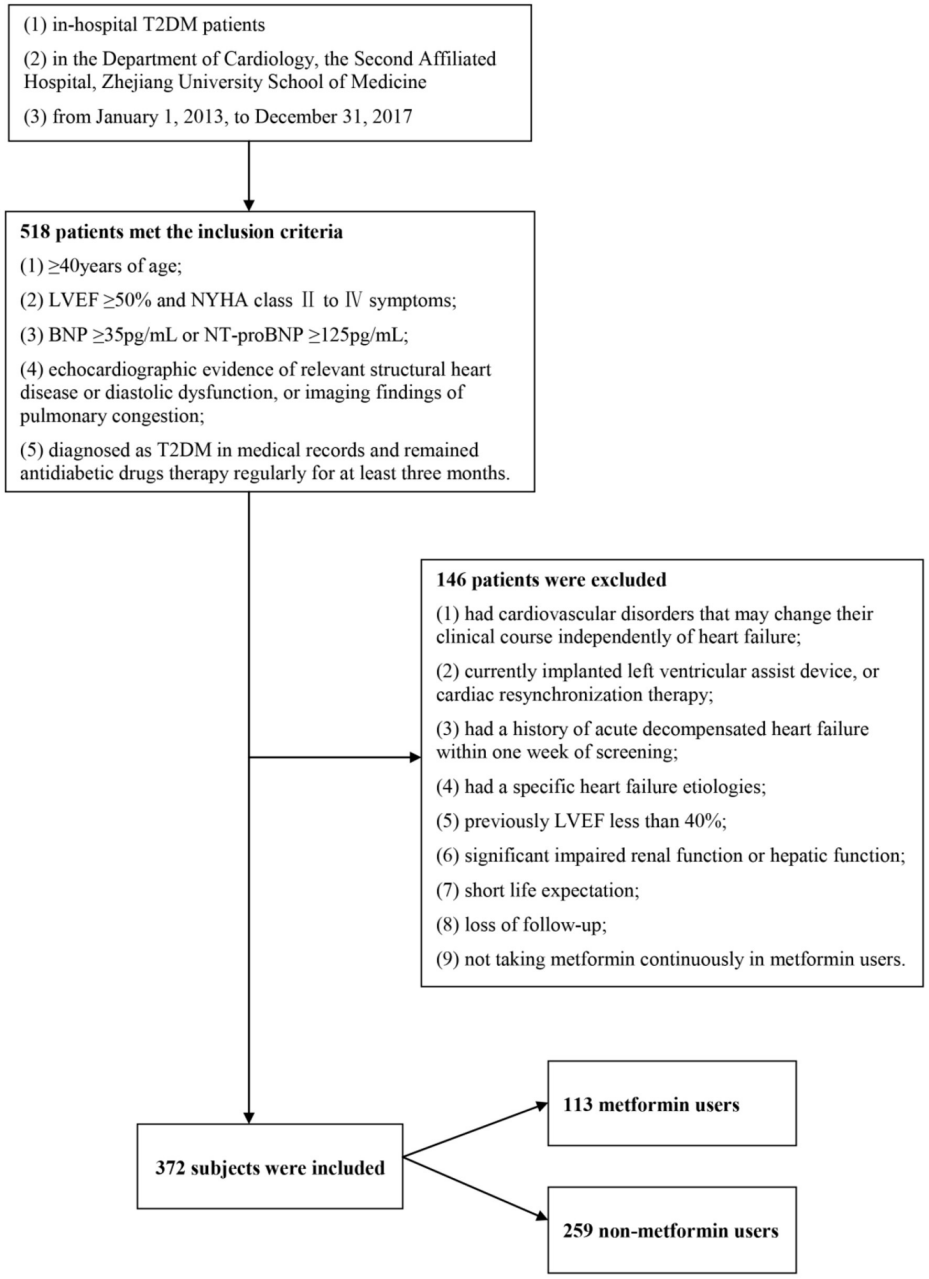
Study population. Among the 518 subjects, we excluded 146 subjects who did not meet our inclusion criteria. The remaining 372 subjects were divided into the metformin group (*n* = 113) and the non-metformin group (*n* = 259).

Among the 518 subjects, we excluded 146 subjects who did not meet our inclusion criteria. The remaining 372 subjects were divided into the metformin group (*n* = 113) and the non-metformin group (*n* = 259) (Figure 1). The median age was 71 years old (interquartile range, 65–79), and 52.4% were male gender. 26.6% of the subjects were categorized as NYHA class III-IV, and few patients have taken thiazolidinediones (TZD) or dipeptidyl peptidase-4 (DPP4) inhibitor. Major imbalances were spotted in age (*P* = 0.000), gender (*P* = 0.001), diastolic blood pressure (*P* = 0.001), LVEF (*P* = 0.021), eGFR (*P* = 0.000) and glucosidase inhibitor usage (*P* = 0.001) between metformin and non-metformin users (Table 1). The date of the last patient follow-up was August 2, 2020. The median duration of participation in the study was 47 months (interquartile range, 38–67).

**Table 1:** Demographic and clinical characteristics of the patients.

**Characteristics**	**Metformin (*n* = 113)**	**Non-metformin (*n* = 259)**	***P-*value**
**Demographic characteristics**			
Age (yr)	68.00 (62.00, 73.00)	73.00 (66.00, 80.00)	0.000
Male	44 (38.9%)	151 (58.3%)	0.001
Smoking	29 (25.7%)	79 (30.5%)	0.344
BMI (kg/m^2^)	25.26(23.37, 27.51)	25.00(23.01, 27.04)	0.151
SBP (mmHg)	130.00 (119.00, 139.00)	130.00 (120.00, 140.00)	0.858
DBP (mmHg)	75.00 (67.00, 83.00)	71.00 (63.00, 78.00)	0.001
NYHA class III-IV	26 (23.0%)	73 (28.2%)	0.299
LVEF (%)	64.97 ± 7.24	63.09 ± 7.14	0.021
**Underlying diseases**			
DM duration (yr)	10.00 (5.00, 10.00)	8.00 (4.00, 10.00)	0.280
HF duration (yr)	1.00 (0.10, 5.00)	1.00 (0.17, 5.00)	0.808
HTN	94 (83.2%)	214 (82.6%)	0.895
CHD	64 (56.6%)	173 (66.8%)	0.061
AF	49 (43.4%)	98 (37.8%)	0.316
CI	14 (12.4%)	37 (14.3%)	0.625
**Laboratory reports**			
HbA1c (%)	7.20 (6.50, 8.35)	7.30 (6.60, 8.46)	0.782
eGFR (ml/min/1.73 m^2^)	91.53 (77.11, 100.24)	81.55 (66.55, 93.32)	0.000
BNP (pg/mL)	84.55 (47.85, 171.63)	104.90 (57.25, 240.10)	0.074
NT-proBNP (pg/mL)	543.00 (305.00, 1470.00)	999.00 (324.00, 2194.00)	0.303
Hb (g/L)	125.00 (117.50, 133.00)	128.00 (117.00, 137.00)	0.169
LDL (mmol/L)	1.89 (1.39, 2.61)	2.02 (1.56, 2.57)	0.212
HDL (mmol/L)	1.06 (0.89, 1.23)	1.08 (0.92, 1.23)	0.417
TG (mmol/L)	1.43 (1.07, 1.96)	1.29 (0.91, 1.78)	0.081
LA (cm)	3.97 (3.70, 4.36)	4.11 (3.80, 4.53)	0.103
**Antidiabetic therapy**			
Sulfonylureas	43 (38.1%)	134 (36.0%)	0.590
Glinides	18 (15.9%)	47 (18.1%)	0.604
GI	158 (61.0%)	48 (42.5%)	0.001
TZD	4 (3.5%)	10 (3.9%)	0.881
DPP4 inhibitor	3 (2.7%)	9 (3.5%)	0.681
Insulin	25 (22.1%)	80 (30.9%)	0.084

*Values are means ± standard deviation or median (interquartile range) or the number of participants (%). AF, atrial fibrillation; BMI, body mass index; BNP, B-type natriuretic peptide; CHD, coronary heart disease; CI, cerebral infarction; DBP, diastolic blood pressure; DM, diabetes mellitus; DPP4, dipeptidyl peptidase-4; eGFR, estimated glomerular filtration rate; GI, glucosidase inhibitors; Hb, hemoglobin; HbA1c, glycated hemoglobin; HDL-C, high-density lipoprotein cholesterol; HF, heart failure; HTN, hypertension; LA, left atrial/atrium; LDL-C, low-density lipoprotein cholesterol; LVEF, left ventricular ejection fraction; NT-proBNP, N-terminal pro-B-type natriuretic peptide; NYHA, New York Heart Association; SBP, systemic blood pressure; TG, triglyceride; TZD, thiazolidinediones*.

### Association Between Metformin and Clinical Outcomes

**Figure 2:**
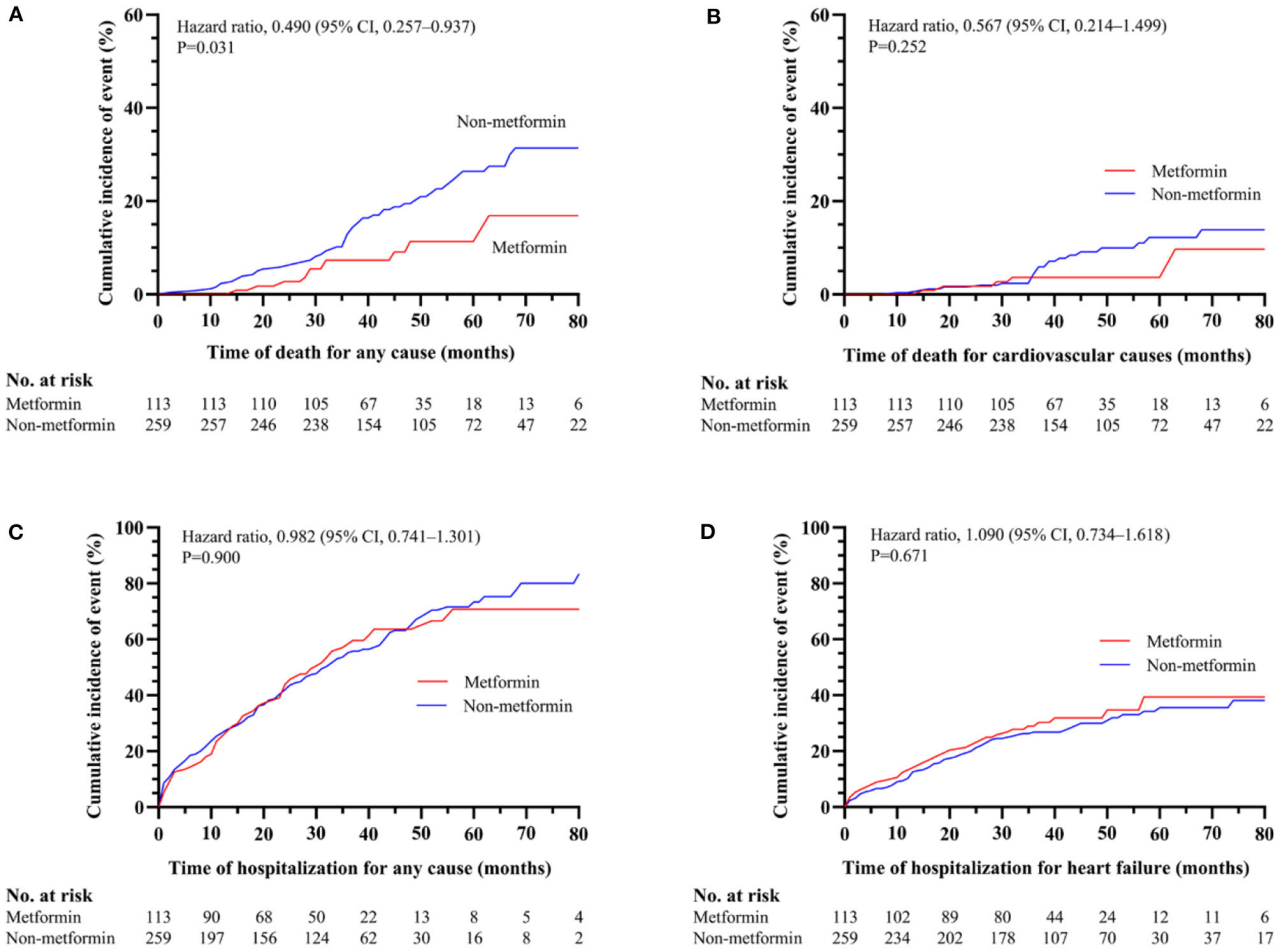
The association between metformin and non-metformin and clinical outcomes in the overall cohort. Metformin was positively related to all-cause mortality (*P* = 0.031) **(A)**. However, metformin has no effect on cardiovascular death (*P* = 0.252) **(B)**, all-cause hospitalizations (*P* = 0.900) **(C)** or hospitalization for heart failure (*P* = 0.671) **(D)**.

In current study, 11 patients (2.49% per patient-year) in metformin group and 56 patients (5.52% per patient-year) in non-metformin group deceased during follow-up. The 1-, 2-, 3-, and 4-year survival rates of the metformin group were 100, 97.3, 92.7, and 88.7%, and the non-metformin group were 97.7, 94.2, 87.1, and 80.5%, respectively, with statistically significant differences (*P* = 0.031) (Figure 2A). There was no statistically significant association between metformin and cardiovascular death (*P* = 0.252), all-cause hospitalizations (*P* = 0.900), and hospitalization for heart failure (*P* = 0.671) (Figures 2B–D and Table 2). However, a multivariable Cox regression failed to show that metformin was an independent factor of all-cause mortality [HR (95% CI) = 0.682 (0.346–1.345); *P* = 0.269] (Table 2).

**Table 2:** The association between metformin and endpoints.

	**Metformin**	**Non-metformin**	**Hazard ratio**	***P-*value**
	**(*n* = 113)**	**(*n* = 259)**	**(95% CI)**	
All-cause mortality^*^	11 (2.49)	56 (5.52)	0.682 (0.346, 1.345)	0.269
Cardiovascular death^†^	5 (1.13)	22 (2.17)	0.567 (0.214, 1.499)	0.252
All-cause hospitalizations^†^	69 (15.59)	165 (16.27)	0.982 (0.741, 1.301)	0.900
Hospitalization for heart failure^†^	36 (8.13%)	78 (7.69)	1.090 (0.734, 1.618)	0.671

*Data were presented as no. of patients with events (% per patient-year)*.

* *For all-cause mortality, hazard ratios and 95% CIs were estimated using Cox regression models. Adjusted covariables included age, gender, body mass index, cigarette smoking, systemic blood pressure, diastolic blood pressure, New York Heart Association class, left ventricular ejection fraction, duration of diabetes and heart failure, whether living with hypertension, atrial fibrillation, coronary heart disease, or cerebral infarction, glycated hemoglobin, estimated glomerular filtration rate, hemoglobin, low-density lipoprotein cholesterol, high-density lipoprotein cholesterol, triglyceride, left atrial, sulfonylureas, glinides, glucosidase inhibitors, insulin*.

† *Hazard ratios and 95% CIs for secondary outcomes were not adjusted for multiplicity*.

### Association Between Metformin and Primary Outcome in Selected Subgroups for Multiplicity

**Figure 3:**
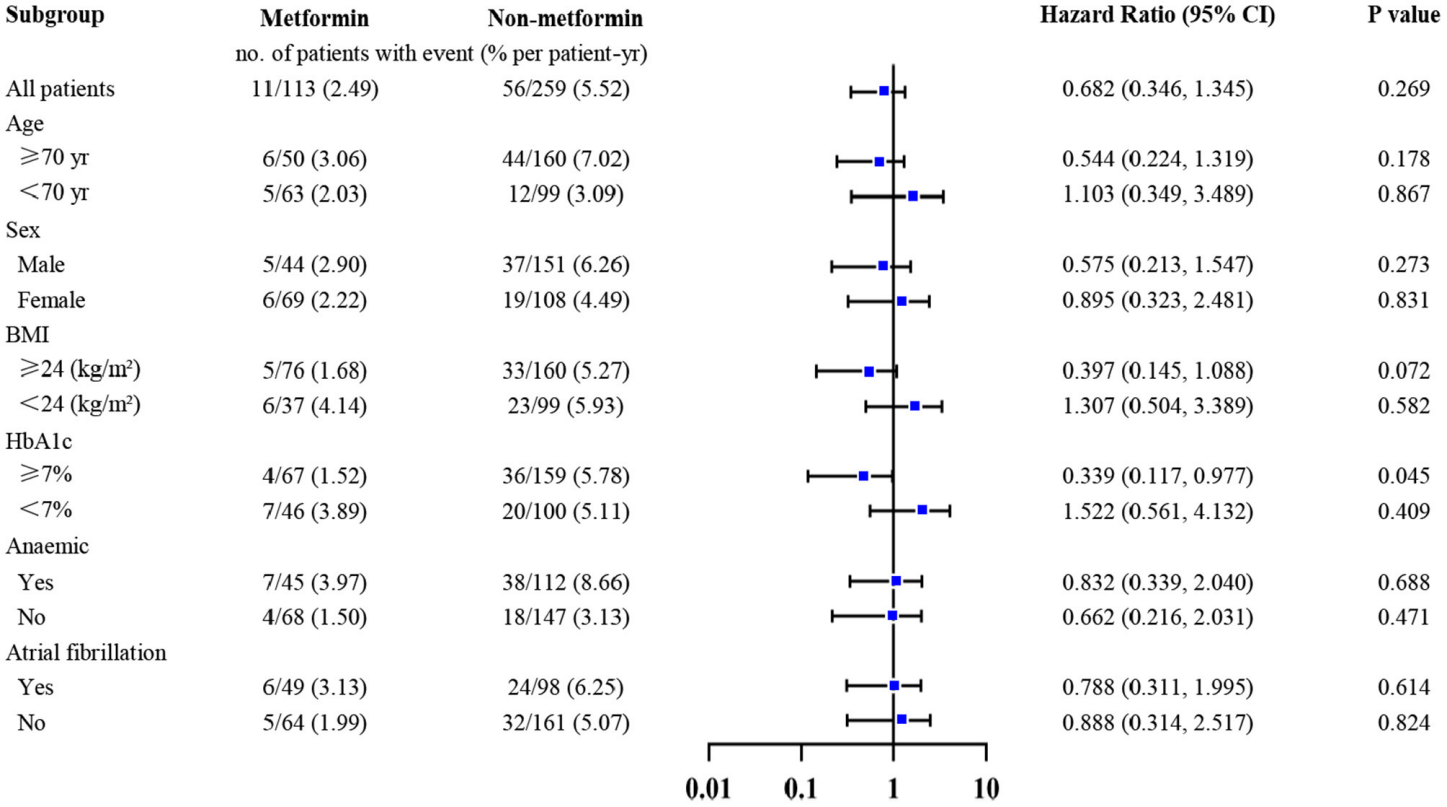
Association between metformin and all-cause mortality in selected subgroups adjusted for multiplicity. Metformin was only associated with lower all-cause mortality in patients whose HbA1c ≥7% [HR (95% CI) = 0.339 (0.117–0.977); *P* = 0.045]. The widths of the confidence intervals were adjusted for multiplicity, using the stepwise regression, with a threshold of 0.1. Adjusted covariables included age, gender, body mass index, cigarette smoking, systemic blood pressure, diastolic blood pressure, New York Heart Association class, left ventricular ejection fraction, duration of diabetes and heart failure, whether living with hypertension, atrial fibrillation, coronary heart disease, or cerebral infarction, glycated hemoglobin, estimated glomerular filtration rate, hemoglobin, low-density lipoprotein cholesterol, high-density lipoprotein cholesterol, triglyceride, left atrial, sulfonylureas, glinides, glucosidase inhibitors, insulin.

We explored the association between metformin and the primary outcome in selected subgroups (stratified according to age, gender, anemia, atrial fibrillation, level of HbA1c, and BMI), using a Cox proportional-hazards model to adjust the hazard ratio and widths of the confidence intervals, and the results were listed in Figure 3. Notably, metformin usage was associated with lower all-cause mortality only in patients with HbA1c ≥7% [HR (95% CI) = 0.339 (0.117–0.977); *P* = 0.045].

### Association Between Metformin Usage and Clinical Outcomes in Subjects With HbA1c ≥7%

**Figure 4:**
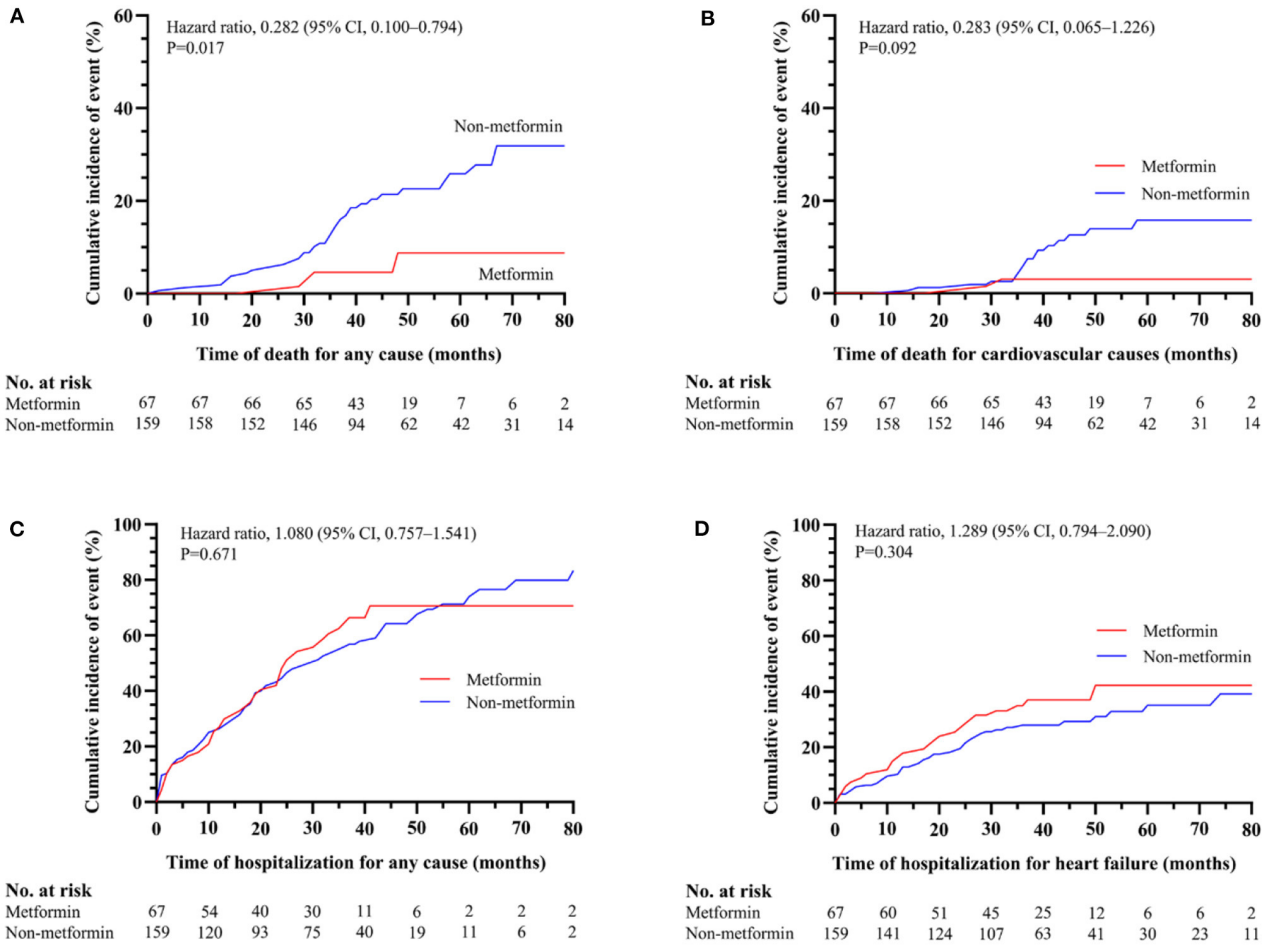
The association between metformin and non-metformin and clinical outcomes in HbA1c ≥7% subgroup. Metformin was positively related to the all-cause mortality (*P* = 0.017) **(A)** but has no effect on cardiovascular death (*P* = 0.092) **(B)**, all-cause hospitalizations (*P* = 0.671) **(C)**, or hospitalization for heart failure (*P* = 0.304) **(D)**.

The demographic and clinical characteristics of subjects with HbA1c ≥7% are described in Table 3. Among the 226 subjects in the HbA1C>7% subgroup, 156 subjects were included for further analysis after PSM. The significant differences between the metformin and non-metformin groups (i.e., differences in age, sex, eGFR, and GI treatment) were adjusted after match. Multivariable Cox regression analysis demonstrated that metformin usage was significantly associated with lower all-cause mortality before match [HR (95% CI) = 0.339 (0.117–0.977); *P* = 0.045] (Table 4). The associations remained unchanged after PSM [HR (95% CI) = 0.292 (0.093–0.913); *P* = 0.034] (Table 5). Same as the results in the full cohort, there was no independent association between metformin use and secondary outcomes in the HbA1c ≥7% subgroup (Figure 4 and Tables 4, 5).

**Table 3:** Demographic and clinical characteristics of the patients in the HbA1c ≥7% subgroup.

**Variables**	**Metformin usage (before PSM)**			**Metformin usage (after PSM)**		
	**Metformin**	**Non-metformin**	***P-*value**	**Metformin**	**Non-metformin**	***P-*value**
	**(*n* = 67)**	**(*n* = 159)**		**(*n* = 60)**	**(*n* = 96)**	
**Demographic characteristics**						
Age (yr)	69.00 (63.00, 73.00)	74.00 (66.00, 80.00)	0.000	69.00 (64.00, 74.00)	70.00 (65.00, 75.75)	0.295
Male	28 (41.8%)	89 (56.0%)	0.051	27 (45.0%)	41 (42.7%)	0.779
Smoking	20 (29.9%)	48 (30.2%)	0.960	19 (31.7%)	24 (25.0%)	0.369
BMI(kg/m^2^)	25.35 (23.73, 27.63)	24.80 (22.68, 27.12)	0.043	25.31 (23.75, 27.95)	24.85 (22.67, 27.38)	0.096
SBP(mmHg)	126.00 (117.00, 139.00)	131.00 (119.00, 140.00)	0.206	126.00 (119.00, 138.75)	130.50 (119.25, 140.00)	0.291
DBP(mmHg)	74.00 (66.00, 81.00)	71.00 (63.00, 79.00)	0.263	73.50 (63.75, 80.75)	72.00 (64.00, 79.00)	0.658
NYHA class III-IV	17 (25.4%)	42 (26.4%)	0.871	16 (26.7%)	23 (24.0%)	0.709
LVEF(%)	64.42 ± 7.24	63.19 ± 7.33	0.249	64.45 ± 7.42	63.42 ± 6.64	0.410
**Underlying diseases**						
DM duration (yr)	10.00 (5.00, 14.00)	10.00 (5.00, 13.00)	0.262	10.00 (5.00, 16.25)	8.00 (4.00, 14.00)	0.131
HF duration (yr)	1.00 (0.10, 6.00)	1.00 (0.10, 4.00)	0.680	2.00 (0.17, 6.00)	1.00 (0.10, 5.00)	0.533
HTN	56 (83.6%)	127 (79.9%)	0.517	50 (83.3%)	74 (77.1%)	0.347
CHD	39 (58.2%)	105 (66.0%)	0.264	36 (60.0%)	61 (63.5%)	0.657
AF	27 (40.3%)	64 (40.3%)	0.995	22 (36.7%)	38 (39.6%)	0.716
CI	8 (11.9%)	24 (15.1%)	0.535	8 (13.3%)	10 (10.4%)	0.579
**Laboratory reports**						
HbA1C (%)	8.00 (7.50, 8.90)	8.10 (7.40, 9.10)	0.547	8.00 (7.50, 9.00)	8.15 (7.50, 9.19)	0.406
eGFR (ml/min/1.73 m^2^)	89.10 (76.27, 98.64)	82.49 (66.75, 94.16)	0.034	88.97 (74.96, 99.00)	86.73 (68.86, 95.47)	0.382
BNP (pg/mL)	84.55 (44.20, 174.75)	117.75 (57.08, 268.63)	0.711	84.90 (43.80, 175.20)	115.60 (56.40, 312.25)	0.094
NT-proBNP (pg/mL)	645.00 (404.00, 1869.00)	1009.50 (327.75, 2199.25)	0.273	703.00 (430.00, 1969.50)	890.00 (266.75, 2197.75)	0.767
Hb (g/L)	125.00 (119.00, 133.00)	128.00 (118.00, 137.00)	0.372	126.00 (117.00, 139.00)	128.50 (118.00, 137.00)	0.345
LDL (mmol/L)	1.84 (1.33, 2.52)	2.10 (1.59, 2.73)	0.765	1.87 (1.34, 2.51)	2.14 (1.64, 2.84)	0.030
HDL (mmol/L)	1.09(0.90, 1.24)	1.08(0.92, 1.22)	0.934	1.08(0.89, 1.23)	1.10(0.94, 1.22)	0.727
TG (mmol/L)	1.32 (1.03, 1.83)	1.31 (0.91, 1.70)	0.341	1.39 (1.06, 1.92)	1.39 (0.92, 1.85)	0.597
LA (cm)	3.95 (3.72, 4.32)	4.04 (3.75, 4.51)	0.377	3.96 (3.73, 4.34)	4.05 (3.71, 4.53)	0.699
**Antidiabetic therapy**						
Sulfonylureas	25 (37.3%)	52 (32.7%)	0.504	22 (36.7%)	28 (29.2%)	0.329
Glinides	10 (14.9%)	29 (18.2%)	0.547	10 (16.7%)	15 (15.6%)	0.863
GI	33 (49.3%)	108 (67.9%)	0.008	30 (50.0%)	60 (62.5%)	0.124
TZD	1 (1.5%)	7 (4.4%)	0.280	0 (0%)	5 (5.2%)	0.072
DPP4 inhibitor	2 (3.0%)	8 (5.0%)	0.494	2 (3.3%)	5 (5.2%)	0.582
Insulin	21 (31.3%)	58 (36.5%)	0.460	19 (31.7%)	39 (40.6%)	0.260

*Values are means ± standard deviation or median (interquartile range) or number of participants (%). AF, atrial fibrillation; BMI, body mass index; BNP, B-type natriuretic peptide; CHD, coronary heart disease; CI, cerebral infarction; DBP, diastolic blood pressure; DM, diabetes mellitus; DPP4, dipeptidyl peptidase-4; eGFR, estimated glomerular filtration rate; GI, glucosidase inhibitors; Hb, hemoglobin; HbA1c, glycated hemoglobin; HDL-C, high-density lipoprotein cholesterol; HF, heart failure; HTN, hypertension; LA, left atrial/atrium; LDL-C, low-density lipoprotein cholesterol; LVEF, left ventricular ejection fraction; NT-proBNP, N-terminal pro-B-type natriuretic peptide; NYHA, New York Heart Association; SBP, systemic blood pressure; TG, triglyceride; TZD, thiazolidinediones*.

**Table 4:** The association between metformin and endpoints in HbA1c ≥7% subgroup before PSM.

	**Metformin**	**Non-metformin**	**Hazard ratio**	***P*-value**
	**(*n* = 67)**	**(*n* = 159)**	**(95% CI)**	
All-cause mortality^*^	4 (1.52)	36 (5.78)	0.339 (0.117, 0.977)	0.045
Cardiovascular death^†^	2 (0.76)	17 (2.73)	0.283 (0.065, 1.226)	0.092
All-cause hospitalizations^†^	44 (16.77)	103 (16.54)	1.080 (0.757, 1.541)	0.671
Hospitalization for heart failure^†^	25 (9.53)	49 (7.87)	1.289 (0.794, 2.090)	0.304

*PSM, propensity score matching*.

*Data were presented as no. of patients with events (% per patient-year)*.

* *For all-cause mortality, hazard ratios and 95% CIs were estimated using Cox regression models, stratified according to level of HbA1c. Adjusted covariables included age, gender, body mass index, cigarette smoking, systemic blood pressure, diastolic blood pressure, New York Heart Association class, left ventricular ejection fraction, duration of diabetes and heart failure, whether living with hypertension, atrial fibrillation, coronary heart disease, or cerebral infarction, glycated hemoglobin, estimated glomerular filtration rate, hemoglobin, low-density lipoprotein cholesterol, high-density lipoprotein cholesterol, triglyceride, left atrial, sulfonylureas, glinides, glucosidase inhibitors, insulin*.

† *Hazard ratios and 95% CIs for secondary outcomes were not adjusted for multiplicity*.

**Table 5:** The association between metformin and endpoints in HbA1c ≥7% subgroup after PSM.

	**Metformin**	**Non-metformin**	**Hazard ratio**	***P*-value**
	**(*n* = 60)**	**(*n* = 96)**	**(95% CI)**	
All-cause mortality^*^	4 (1.70)	19 (5.05)	0.292 (0.093, 0.913)	0.034
Cardiovascular death^†^	2 (0.85)	8 (2.13)	0.408 (0.774, 1.706)	0.257
All-cause hospitalizations^†^	42 (17.87)	64 (17.02)	1.149 (0.774, 1.706)	0.491
Hospitalization for heart failure^†^	23 (9.79)	31 (8.24)	1.267 (0.735, 2.184)	0.394

*PSM, propensity score matching*.

*Data were presented as no. of patients with events (% per patient-year)*.

* *For all-cause mortality, hazard ratios and 95% CIs were estimated using Cox regression models, stratified according to level of HbA1c. Adjusted covariables included age, gender, body mass index, cigarette smoking, systemic blood pressure, diastolic blood pressure, New York Heart Association class, left ventricular ejection fraction, duration of diabetes and heart failure, whether living with hypertension, atrial fibrillation, coronary heart disease, or cerebral infarction, glycated hemoglobin, estimated glomerular filtration rate, hemoglobin, low-density lipoprotein cholesterol, high-density lipoprotein cholesterol, triglyceride, left atrial, sulfonylureas, glinides, glucosidase inhibitors, insulin*.

† *Hazard ratios and 95% CIs for secondary outcomes were not adjusted for multiplicity*.

### Absolute Risk Reduction of All-Cause Mortality With Metformin in Comparison With Non-metformin

Cumulative incidence of event, incident rates, relative risk reduction, and number needed to treat (NNT) values for the overall cohort and the HbA1c ≥7% subgroup by year are displayed in Table 6. The 4-year estimated NNT with metformin compared with non-metformin for all-cause mortality was 12 in all populations and 8 in the HbA1c ≥7% subgroup.

**Table 6:** Event rates, incidence rates, and number needed to treat for all-cause mortality for comparison of metformin with non-metformin.

	**All-cause mortality in whole population (*n* = 372)**	**All-cause mortality in the HbA1c ≥7% subgroup (*n* = 226)**
**Events/total number**		
Metformin	11/113	4/67
Non-metformin	56/259	36/159
*P-*value^*^	0.269	0.045
**Incidence rate, %**		
Metformin	9.7	6
Non-metformin	21.6	22.6
Difference	11.9	16.6
**Relative risk reduction, %**	55	73
**Number needed to treat**		
1-year	44	46
2-year	32	20
3-year	20	10
4-year	12	8

* *Events of all-cause mortality were estimated using Cox regression models. Adjusted covariables included age, gender, body mass index, cigarette smoking, systemic blood pressure, diastolic blood pressure, New York Heart Association class, left ventricular ejection fraction, duration of diabetes and heart failure, whether living with hypertension, atrial fibrillation, coronary heart disease, or cerebral infarction, glycated hemoglobin, estimated glomerular filtration rate, hemoglobin, low-density lipoprotein cholesterol, high-density lipoprotein cholesterol, triglyceride, left atrial, sulfonylureas, glinides, glucosidase inhibitors, insulin*.

## Discussion

In the present study, we investigated the association between metformin and adverse outcome on HFpEF with T2DM populations. The major finding was that metformin was linked to a lower incidence of death from any causes in HFpEF and T2DM patients with poor glucose control.

Tremendous advancements have been made in the treatment of HFrEF employing neurohumoral activation. Nevertheless, no therapy has been shown to reduce morbidity or mortality in HFpEF patients (4). Furthermore, the pathophysiology underlying HFpEF is heterogeneous and is associated with multiple phenotypes, including cardiovascular and non-cardiovascular comorbidities (2). The cause of death and hospitalization among HFpEF patients is more likely to be non-cardiovascular than patients with HFrEF (10). Thus, management of comorbidity is an essential task for HFpEF patients.

Compared with the general population, diabetes doubles and quintuples HF's risk in males and females, respectively (11). About 45% of HFpEF patients suffer from diabetes (1). Diabetic patients tended to combine with structural and functional echocardiographic abnormalities (6). Clinical trial data suggest that among individuals with HFpEF, those with diabetes were associated with worse health-related quality of life and increased risk of hospitalization, cardiovascular mortality, and all-cause mortality (6, 12).

The most critical finding in the present study is that metformin reduced all-cause mortality in HFpEF and T2DM patients with poor glucose control. Metformin treatment improved glycaemic and reduced cardiovascular mortality, without the risk of hypoglycemia or bodyweight gains associated with the use of other antidiabetic drugs (13–15). Therefore, it is currently the preferred oral antidiabetics in T2DM and heart failure patients (16). A recent study showed that long-term prescription of metformin could improve left ventricular diastolic function and delay the progression of HFpEF in T2DM and hypertension population (17). Slater et al. reported that metformin improves diastolic function in a mouse model with HFpEF-like symptoms by lowering titin-based passive stiffness (18). The mechanisms by which metformin exerts favorable effects of metformin on HF progression are still not fully understood. Adenosine monophosphate-activated protein kinase (AMPK) is correlated with cardiac fatty acids uptake, autophagy, mitochondrial biogenesis, and energy regulation. Changes in the adenosine AMPK pathway play a major role in developing myocardial impairment (19, 20). In addition to glycaemic control, metformin appears pleiotropic effects. In a diabetic heart, metformin regulates lipid and glucose metabolism *via* AMPK activation and further improves cardiac energy metabolism (20). What's more, metformin can improve mitochondria function, increasing nitric oxide bioavailability, inhibit the interstitial accumulation of collagen and cardiomyocyte apoptosis through AMPK-dependent or AMPK-independent pathways, and thereby reduce cardiac remodeling and hypertrophy, and preserve cardiac function (7, 20).

It is worth noting that metformin was linked to a lower incidence of all-cause mortality only in patients with poor glycemic control. Prolonged exposure to pronounced hyperglycemia may have a more sustainable myocardial damage than the lower glucose status, therefore increasing the relative adverse influence of hyperglycemia in HF patients. This might explain why metformin showed a more significant cardioprotective effect in those with poor glycemic control. Finally, a higher HbA1c level means a higher glucose status, a sign of insufficient insulin effect or the underlying insulin resistance. Nevertheless, the specific underlying mechanism needs to be clarified.

Historically, metformin should not be used in patients with heart failure due to lactic acidosis risk (21). Nowadays, clinical observations and experimental studies have provided increasing evidence of the safety and benefits of metformin in patients with diabetes and heart failure. A systematic review of observational studies indicates that metformin can be safely used in patients with diabetes mellitus and HF, even in heart failure with reduced left ventricular ejection fraction or chronic kidney failure. Meanwhile, none of the trials demonstrate that metformin was associated with an increased risk of lactic acidosis than other hypoglycemic agents (8).

Several limitations of our study should be acknowledged. First, our study was retrospective rather than randomized prospectively planned, and causality cannot be inferred from these retrospective findings. Second, clinical data were obtained in a single-center instead of multi-centers. Third, the amount of the subject was relatively small. Finally, since our study was conducted before the general introduction of novel antidiabetics, including sodium-glucose co-transporter-2 inhibitors and glucagon-like peptide-1 receptor agonists, they were not included in our analysis.

In conclusion, there was no independent association between metformin use and outcome in the cohort of T2DM with HFpEF. However, metformin was associated with lower all-cause mortality in the subgroup of patients with poor glycemic control. Prospective, large sample studies are necessary to determine the optimal treatment for HFpEF patients with T2DM.

## Data Availability Statement

The raw data supporting the conclusions of this article will be made available by the authors, without undue reservation.

## Ethics Statement

The studies involving human participants were reviewed and approved by Ethics Board of the Second Affiliated Hospital, Zhejiang University School of Medicine. The patients/participants provided their written informed consent to participate in this study.

## Author Contributions

ZC and YL: designed the study. JWa, TY, and XM: contributed data. JWa, YL, and JWe: drafted the manuscript. All authors: were involved in critically revising the manuscript.

## Conflict of Interest

The authors declare that the research was conducted in the absence of any commercial or financial relationships that could be construed as a potential conflict of interest.
